# Editorial: Tropical fungal diseases

**DOI:** 10.3389/fcimb.2022.1104519

**Published:** 2022-12-29

**Authors:** Carlos P. Taborda, Julián Esteban Muñoz, Angel Gonzalez

**Affiliations:** ^1^ Universidade de São Paulo, Instituto de Ciências Biomédicas, Departamento de Microbiologia, São Paulo, Brazil and Instituto de Medicina Tropical, Laboratório de Micologia Médica, LIM53/HCFMUSP, São Paulo, Brazil; ^2^ Studies in Translational Microbiology and Emerging Diseases (MICROS) Research Group, Translational Medicine Institute, School of Medicine and Health Science, Universidad del Rosario, Escuela de Medicina y Ciencias de la Salud, Bogota, Colombia; ^3^ Universidad de Antioquia, Escuela de Microbiología, Medellín, Colombia

**Keywords:** tropical diseases, laboratory diagnostic, antifungal treatment, molecular biology, proteomics

The tropical region of Earth occurs between the latitude lines of the Tropic of Cancer and the Tropic of Capricorn. The tropics include parts of North America, South America, Africa, Asia, and Australia (**Figure 1**). The climate in the tropics is characterized by a more direct sunlight than the rest of Earth, meaning that the tropics are generally hotter and wetter and less affected by the solar seasons.

**Figure 1:**
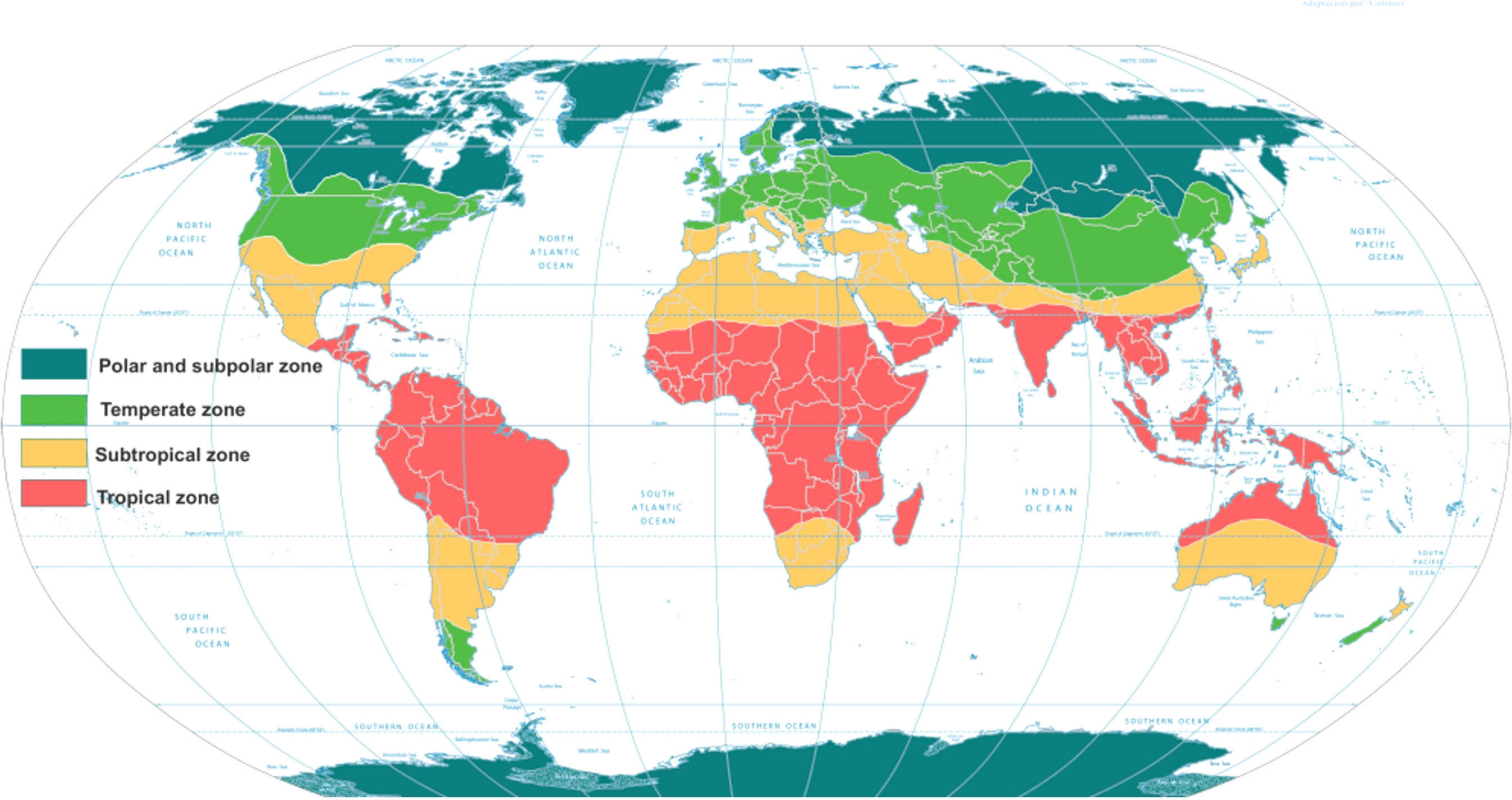
World map with the intertropical zone highlighted in red. (https://content.meteoblue.com/pt/research-education/educational-resources/meteoscool/zonas-climaticas-em-geral).

According to the literature, there are many fungal infections that occur more frequently in tropical zones or are restricted to certain regions within the tropics. The interference in ecosystems mainly by deforestation of tropical areas and re-adaptation of wild animals into the cities has modified the spectrum of the mycoses. Many of these fungal infections, although with important impacts on public health, are still considered neglected and low visibility diseases. The number of reports of immunosuppressed patients with opportunistic fungal diseases has increased significantly across the world. In this special issue, we will explore some of the main or even rare fungal infections that affect tropical areas of the planet. Included among these diseases are cutaneous (dermatophytes or non-dermatophytes), subcutaneous (chromoblastomycosis and sporotrichosis), systemic (cryptococcosis), and endemic (histoplasmosis) mycoses.

Cutaneous mycoses are a group of fungal infections affecting the skin, hair, and nails; however, in patients with autosomal recessive CARF9 deficiency or immunodeficiencies, dermatophytes can cause systemic mycosis (de Oliveira Pereira et al., 2021). The use of tools such as multilocus phylogenetic analysis has distinguished seven main clades: the genera *Arthroderma*, *Lophophyton*, *Microsporum*, *Paraphyton*, *Nannizzia*, *Epidermophyton*, and *Trichophyton* (reviewed by de Oliveira Pereira et al., 2021). Treatment of dermatophytosis is very complex and depends on several factors arising from both the host and the fungus. Drug resistance is a major problem, especially in the treatment of dermatophytosis. Terbinafine is a highly recommended drug to treat dermatophytosis. The determination of the antifungal susceptibility and punctual mutations in isolates of dermatophytes resistant to terbinafine is essential to assist physicians in the management of patients (Pashootan et al.).

Dematiaceous or melanized fungi include a large and heterogeneous group of fungi that cause several diseases including chromoblastomycosis, phaeohyphomycosis, and eumycetoma (reviewed by Arcobello and Revankar, 2020). Phaeohyphomycosis refers to a group of mycoses caused by pigmented fungi characterized by yeast-like cells, hyphae, or a combination of both morphotypes in tissues and should be distinguished from primary implantation mycoses. In the last two decades, the frequency of reports and the diversity of the etiological agents involved in this type of mycosis have increased, especially in immunosuppressed individuals (He et al.). Chromoblastomycosis is one of the most prevalent implantations or subcutaneous fungal infections characterized by traumatic inoculation from an environmental source with clinically polymorphic lesions (Queiroz-Telles et al., 2017). The main agents of Chromoblastomycosis include *Fonsecaea* spp., *Phialophora verrucosa*, *Cladophialophora carrionii*, *Exophiala dermatitidis*, and *Rinocladiella aquaspersa* (Queiroz-Telles et al., 2017). Uncommon agents such as *Chrysosporium keratinophilum*, a saprophytic filamentous fungus commonly found in soil, dung, and animal fur, and rarely involved in infections in humans, was reported in a patient with severe chromoblastomycosis-like lesions (Mijiti et al.).

Genomic studies involving *Fonsecaea* and *Cladophialophora* genera from adverse microhabitats and mammal tissue are extremely important to understand virulence factors. The results of a study suggested a higher level of extremotolerance of environmental species (Vicente et al.). Another study focused on the degree with which *Fonsecaea* agents are involved in pathogenicity. The authors aimed to evaluate a model of trans-kingdom infection. The plant infection models employed suggested that all *Fonsecaea* were saprobic. The authors observed, for the first time, structures similar to muriform cells, in a larvae model, that were produced during infection of human tissue, confirming the role of muriform cells as a pathogenic adaptation in animal tissues (Fornari et al.).

Sporotrichosis, caused by *Sporothrix brasiliensis*, a cat-transmitted fungal infection, is an endemic and neglected mycosis found mainly in Brazil (Gremião et al., 2021). The geographic expansion of this zoonosis has been reported in different regions of Brazil, and more recently, cases were reported in other countries in South America (Gremião et al, 2021). Environmental and epidemiological studies are essential to control the spread of this mycosis. A study conducted in Rio de Janeiro (Brazil), an area highly endemic for feline and human diseases, showed that *S. brasiliensis* can maintain itself persistently in its environment for years. In addition, antifungal susceptibility tests indicated that minimal inhibitory concentration of itraconazole from the environmental isolates was lower with cat isolates; however, with amphotericin B and terbinafine it was similar (Rabello et al.).

In general, invasive fungal diseases are rare in immunocompetent individuals; however, an increasing population of immunocompromised patients and ongoing climate change could significantly increase the prevalence of fungal diseases (Casadevall, 2022). *Histoplasma* spp., the causal agent of histoplasmosis, is an environmental dimorphic fungus with a worldwide distribution. In immunocompetent patients, histoplasmosis usually occurs in a less aggressive form, whereas immunocompromised patients present with a more aggressive clinical form. Hemophagocytic lymphohistiocytosis (HLH) is a rare disorder and is characterized by persistent immune activation of natural killer (NK) and cytotoxic T cells. In this special issue, Chen et al. reviewed cases of HLH secondary to disseminated histoplasmosis in HIV seronegative patients and highlighted that this condition has become increasingly common in emerging endemic areas, carrying a high mortality rate. However, timely diagnosis and early use of antifungals can lead to a favorable prognosis.

Similarly, cryptococcosis is a fungal infection found in immunosuppressed patients, well described in HIV-infected patients, and more rarely occurring in immunocompetent individuals. Goupil de Bouillé et al. described the clinical, mycological, immunological, and genetic characteristics of six HIV-negative patients from French Guiana presenting with invasive cryptococcosis. Despite the available antifungal drugs for cryptococcosis treatment, morbidity and mortality rates remain high. A vaccine would be an important strategy against cryptococcosis infection. Normile and Del Poeta showed in previous studies that a live, attenuated, *Cryptococcus neoformans* Δsgl1 mutant accumulating steryl glucosides was found to be avirulent and protected mice from an otherwise lethal infection. In this study, the validation of three different models of successful vaccination strategies against cryptococcosis are shown, which used heat-killed *C. neoformans* Δsgl1 in a CD4+ T cell deficiency setting.

The contributions described above in this research topic illustrate the changes in behavior and the increase of fungal infections in the tropics, such as the increase in the spread of feline sporotrichosis, the increase in the resistance to antifungals of dermatophytes, and the invasive infection of immunocompetent hosts by “opportunistic” fungi such as *Cryptococcus*, as well as successful advances in experimental vaccines against cryptococcosis. Nonetheless, fungal diseases in tropical and subtropical areas continue to be a public health problem. Most of these fungal diseases are neglected and have low visibility, thereby affecting immunocompromised patients (such as persons living with HIV), people in low-income countries, and particularly agricultural workers in rural areas.

## Author contributions

AG, CT, and JM edited the topic and wrote the manuscript. All authors contributed to the article and approved the submitted version.
